# Supplementary material for: Simulated microgravity confines and fragments the straw-based lignocellulose degrading microbial community
Source: Microbiol Spectr. 2025 Apr 16;13(6):e02466-24. doi: 10.1128/spectrum.02466-24 (PMC12131793; doi:10.1128/spectrum.02466-24)
Supplement: Supplemental material — Tables S1; Fig. S1 to S8. [file spectrum.02466-24-s0001.docx]

**Table S1**. Major topological properties of the empirical networks (pMENs) of mµ-g and 1g and their corresponding random networks.

| Network name | Empirical networks | | | | | | | | Random networks^a^ | | |
| --- | --- | --- | --- | --- | --- | --- | --- | --- | --- | --- | --- |
|  | No. of original ASVs | Similarity threshold | Network size | R^2^ of power law | Average connectivity | Average path distance (GD) | Average clustering coefficient | Modularity (No. of modules) | Average path distance ± SD | Average clustering coefficient ± SD | Average modularity± SD |
|  |  |  |  |  |  |  |  |  |  |  |  |
| mμ-g | 8,265 | 0.88 | 62 | 0.383 | 7.29 | 3.076^b^ | 0.350^b^ | 0.445^b^ | 2.372 +/- 0.045 | 0.229 +/- 0.022 | 0.247 +/- 0.010 |
| 1g | 8,285 | 0.72 | 115 | 0.233 | 18.226 | 2.967^b^ | 0.523^b^ | 0.266^b^ | 2.188 +/- 0.025 | 0.425 +/- 0.018 | 0.116 +/- 0.005 |

^a^The random networks were generated by rewiring all the links of a network with an identical number of nodes and links to the corresponding empirical network.

^b^Significant difference (*p* < 0.050) between empirical networks and random networks.


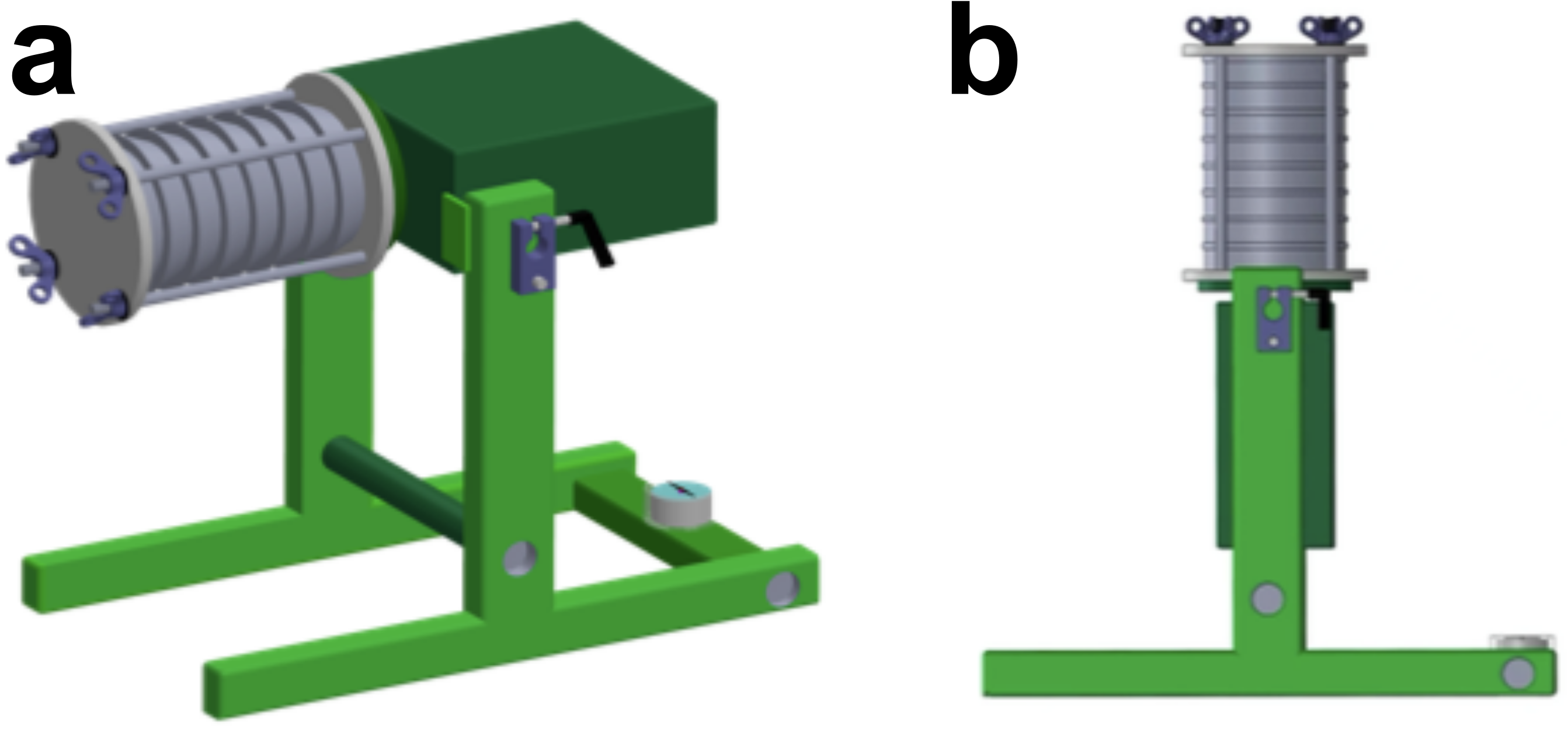


**FIG. S1**. The model diagram of the 2-D random positioning machine (RPM) used to create (a) mµ-g and (b) 1g treatment conditions, with the spinning axes horizontal and vertical, respectively.


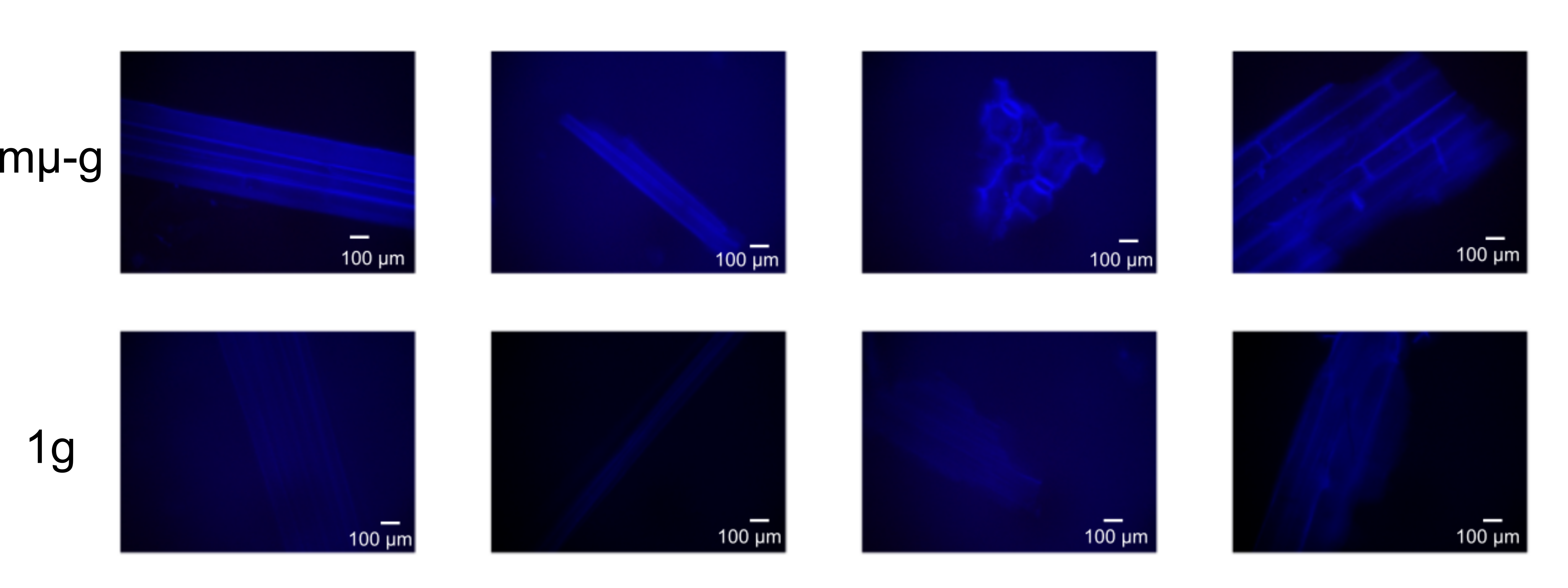


**FIG. S2**. The straw cell wall’s autofluorescence under mµ-g and 1g on the fermentation’s final day (day 25), visualizing the degradation progress.


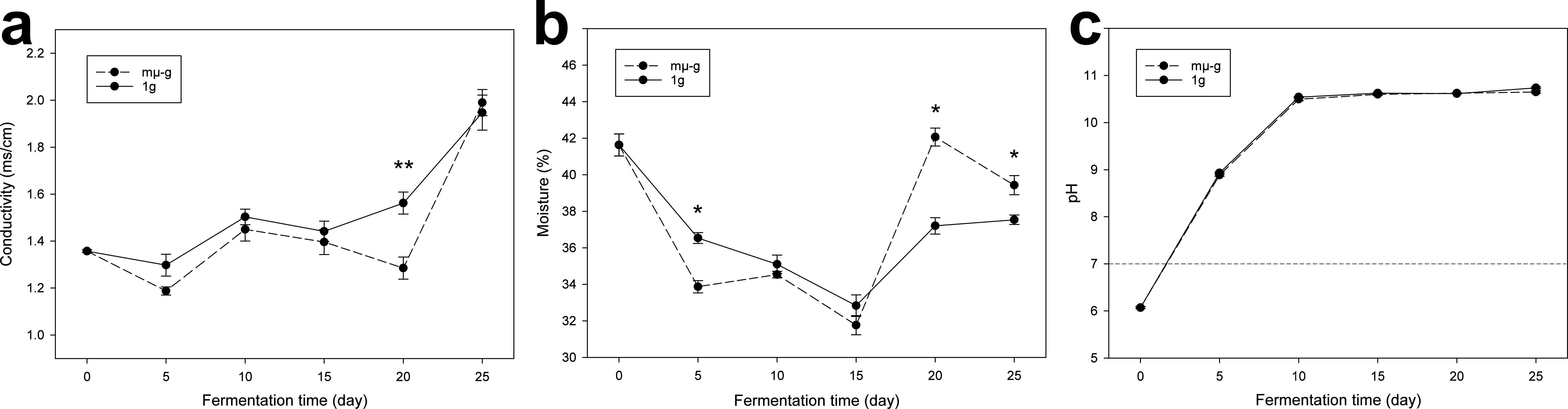


**FIG. S3**. The straw fermentation material’s physical and chemical properties under mµ-g and 1g, including (a) conductivity, (b) moisture, and (c) pH. Error bars represent standard error (*n* = 3 biological replicates). Asterisks indicate significant differences between mµ-g and 1g, as determined by ANOVA (significance marks: *, 0.01≤*P*<0.05; **, 0.001≤*P*<0.01; ***, *P*<0.001).


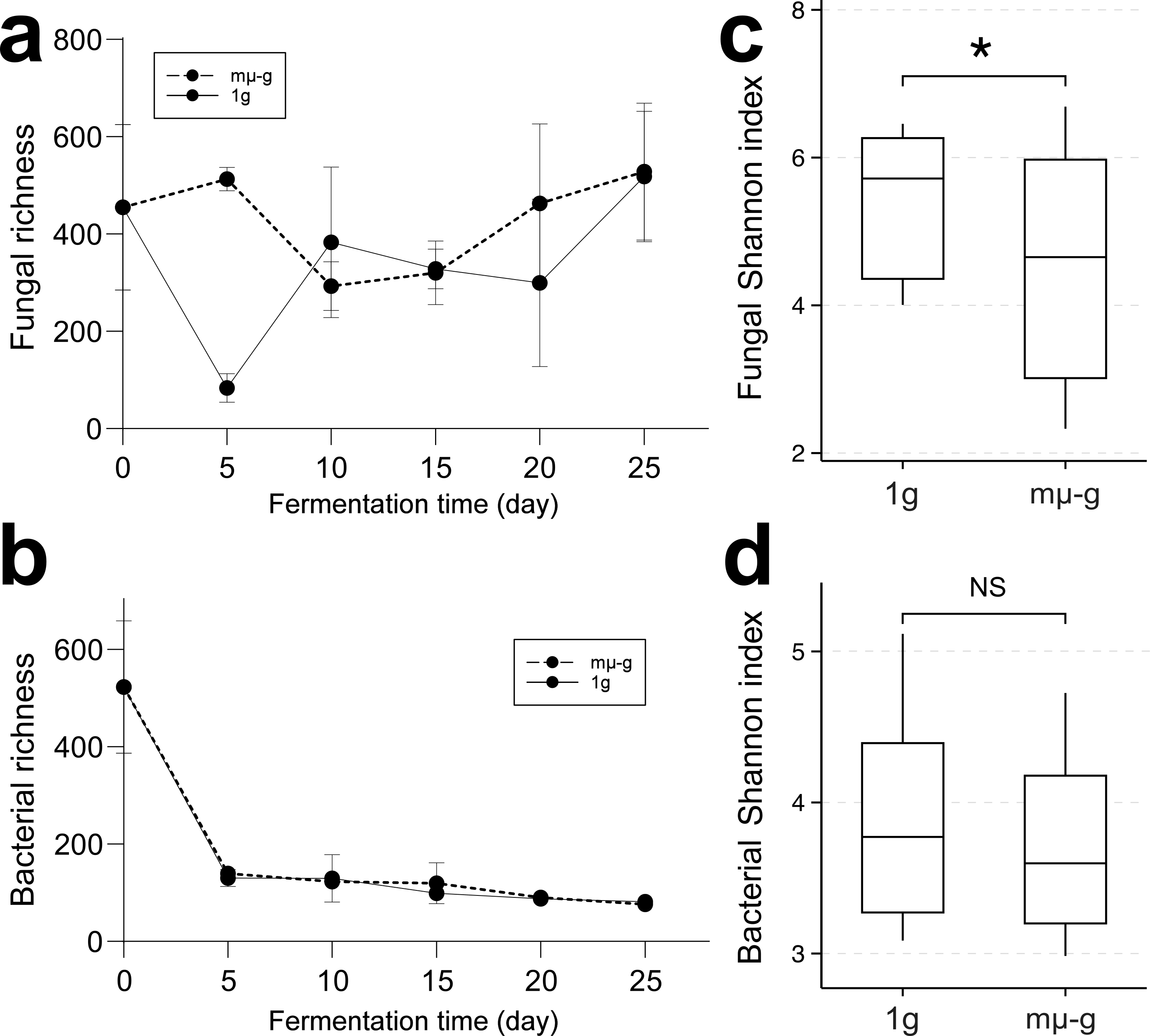


**FIG. S4**. Microbial communities’ diversity indices of the fermentation material, including (a) fungal richness, (b) bacterial richness, (c) fungal Shannon index, and (d) bacterial Shannon index. Error bars represent standard error (*n* = 3 biological replicates). Asterisks indicate significant differences between mµ-g and 1g, as determined by paired student’s *t*-test (significance marks: *, 0.01≤*P*<0.05; **, 0.001≤*P*<0.01; ***, *P*<0.001).


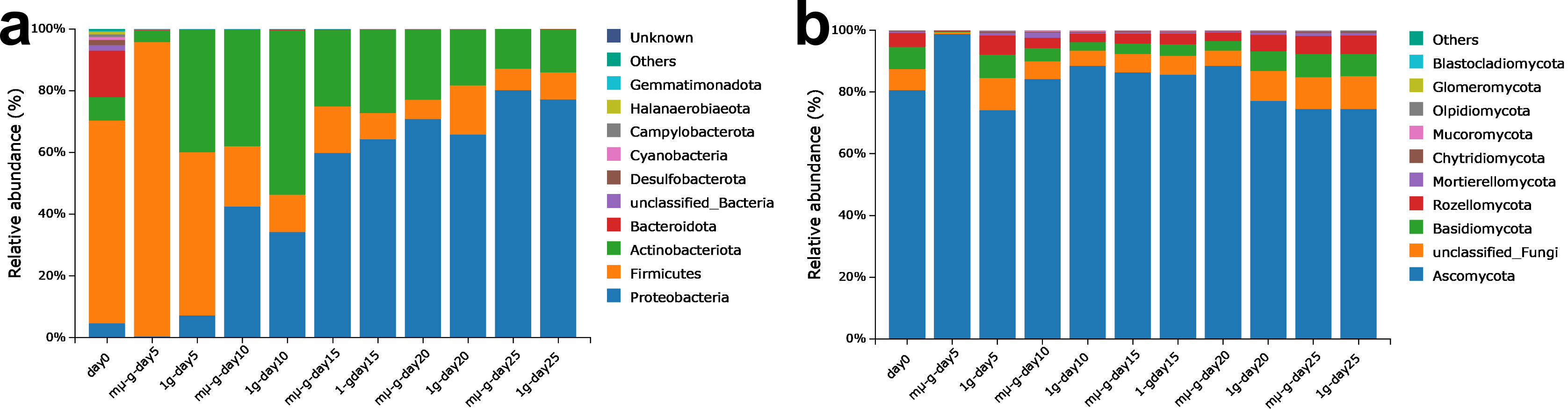


**FIG. S5**. The phylum-level relative abundance of (a) bacterial and (b) fungal microbial communities, as determined by taxonomic annotation.


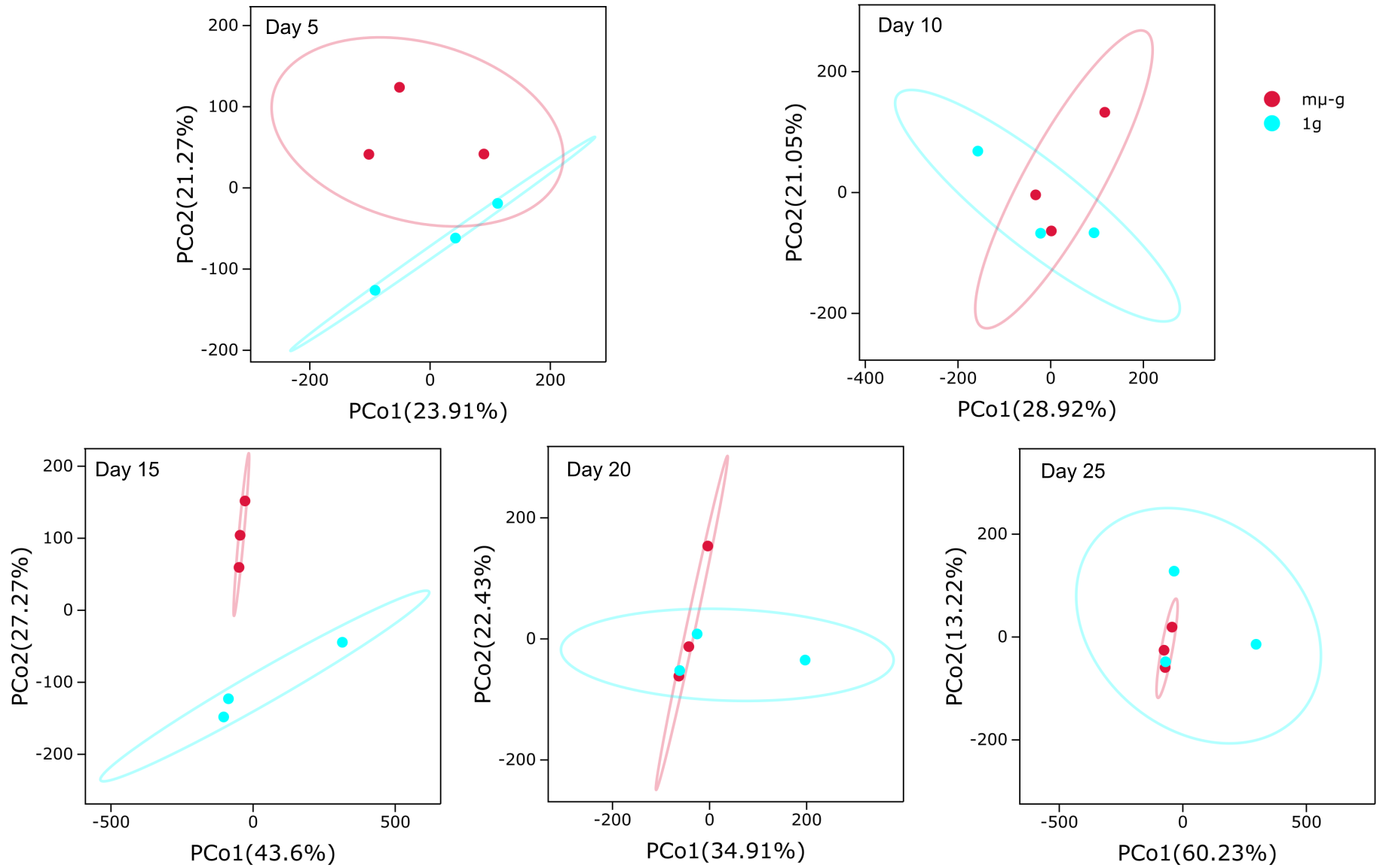


**FIG. S6**. The PCoA depiction of the metabolites’ composition under mµ-g and 1g, of the five sampling time points respectively.

**
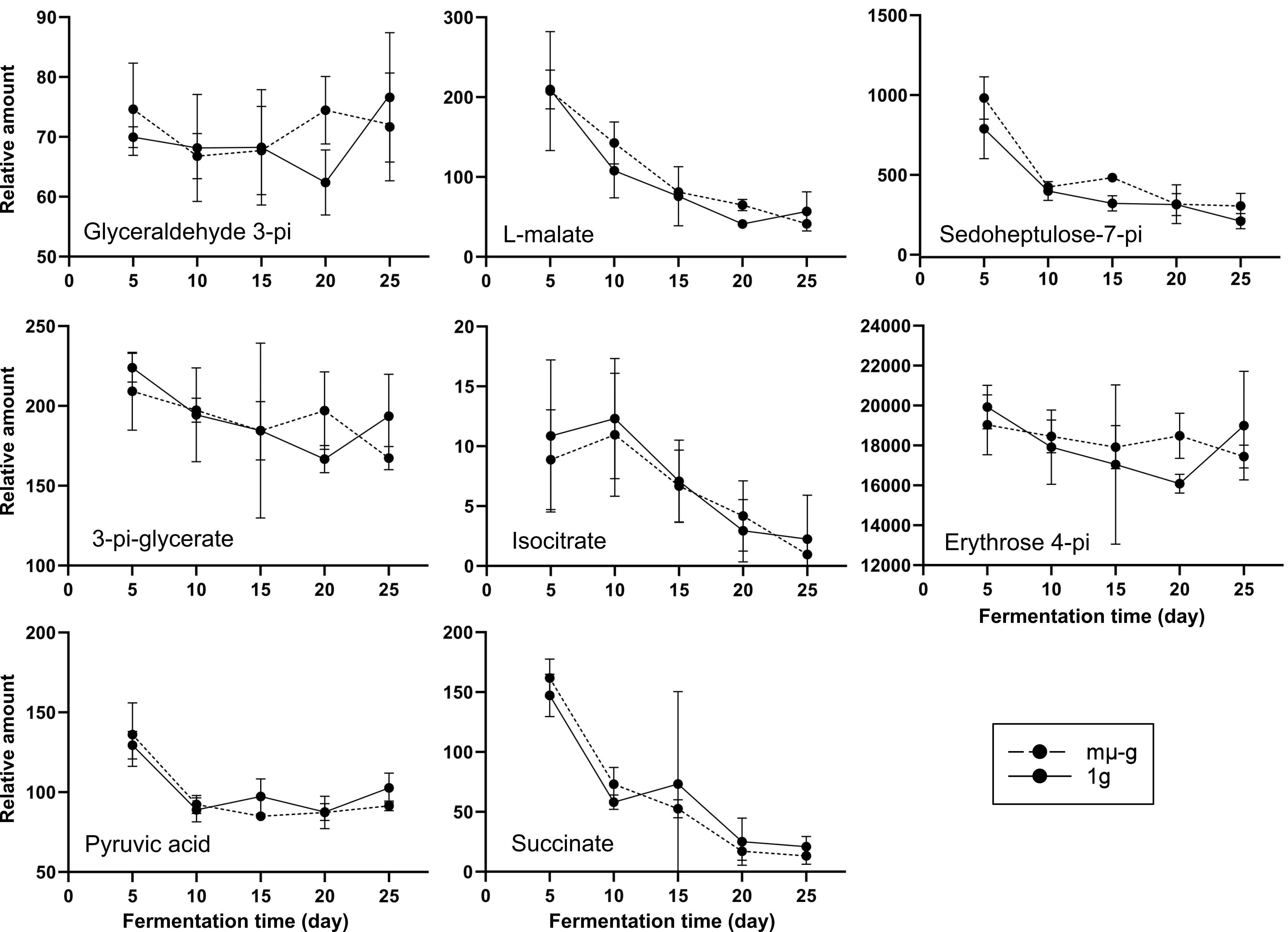
**

**FIG. S7**. The content of intermediate metabolites in three major pathways of glucose metabolism, including glycolysis pathway, tricarboxylic acid cycle and pentose phosphate pathway.


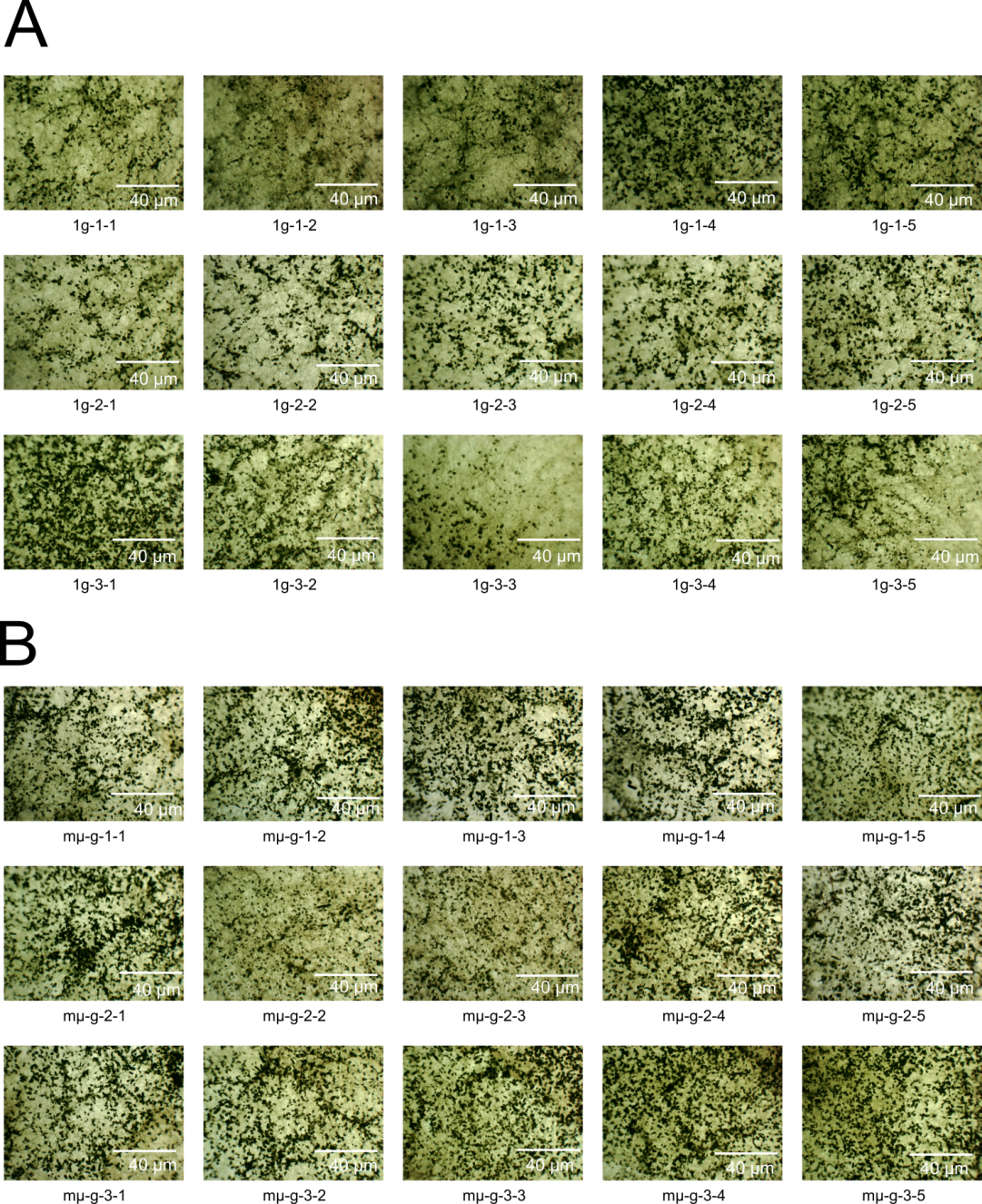


**FIG. S8**. Microscopic observation on the plate pure culture colonies of *Aspergillus nodulans*, showing its ascomata density, under (a) 1g and (b) mµ-g.
